# Minimally Invasive Robot-Guided Dual Cortical Bone Trajectory for Adjacent Segment Disease

**DOI:** 10.7759/cureus.16822

**Published:** 2021-08-02

**Authors:** Kyungduk Rho, Timothy E OConnor, Jean-Marc Lucas, John Pollina, Jeffrey Mullin

**Affiliations:** 1 Neurological Surgery, University at Buffalo, Jacobs School of Medicine and Biomedical Sciences, Buffalo, USA; 2 Osteopathic Medicine, Lake Erie College of Osteopathic Medicine, Bradenton, USA

**Keywords:** robotics, navigation, technology, mazor robot, cortical bone trajectory, stealth, minimally invasive surgery

## Abstract

Here we present a novel application of cortical bone trajectory (CBT) fixation utilizing robotic guidance in a previously instrumented spine with a traditional pedicle screw (PS), obviating the need for a larger posterior incision, reducing the risk of infection, muscular dissection, and likely decreasing hospital length of stay.

A 60-year-old woman with prior left L3-L4 extreme lateral interbody fusion and unilateral percutaneous PS placed at L3 to L5 presented with progressive bilateral lower-extremity pain and diminished sensation in the S1 dermatome secondary to adjacent segment disease (ASD). The patient underwent an L5-S1 anterior lumbar interbody fusion for indirect decompression and restoration of segmental lordosis. After the first stage was completed, she was turned prone for posterior percutaneous instrumentation. Given prior instrumentation at L3-L5 on the left side, a robot planning software was used to plan a cortical bone screw on the left L5 pedicle. A left S1 PS was then planned with the screw head aligning with the left L5 cortical bone screw. Instrumentation was then placed percutaneously using the robot bilaterally without issue. Intraoperative fluoroscopic imaging demonstrated accurate placement of PS, and postoperative computed tomography demonstrated the excellent positioning of all PSs.

This report is the first documented case of a robotically placed CBT screw placed in the same pedicle as a prior traditional PS for ASD. This method expands the surgical options for ASD to include robotic percutaneous placement of posterior instrumentation at the same level as previous instrumentation.

## Introduction

Background and importance

Adjacent segment disease (ASD) is defined as a new degenerative change at a spinal level adjacent to a surgically treated level or levels in the spine with related symptoms, including radiculopathy, myelopathy, or instability [1]. The incidence of lumbar ASD ranges from 2% to 14% of patients, representing a tangible threat to the post-surgery spine patient population [1]. The precise etiology of ASD is unclear, but it has been attributed to changes in intradiscal pressure, misalignment in the sagittal plane, and increased biomechanical stress [2]. Surgical treatment options for ASD include revision posterior procedures; minimally invasive approaches; or indirect decompression with interbody fusions at the anterior face, posterior face, or both [3].

As Mullin et al. demonstrated, cortical bone trajectory (CBT) screws present an alternative form of instrumentation of the posterior spine [4]. CBT screws are medially placed and angled slightly outward in the axial plane, whereas the traditional pedicle screws (PSs) are placed laterally and project inward toward the midline. In the sagittal plane, CBT screws follow a caudocephalad trajectory. These features grant CBT screws greater access to cortical bone, which is dense and strong [5]. Proposed advantages to this approach include reduced likelihood of trauma to the local neurovasculature; increased contact between the screws and the bone, therefore providing a more robust connection; and a less invasive procedure overall given less required muscular dissection [6].

The assistance of imaging technology is required for accurate navigation in placing CBT screws with a minimally invasive approach. Techniques that have been used to successfully guide screw placement include endoscopy, three-dimensional computed tomography (CT), and fluoroscopy [7,8]. All three have shown good accuracy with minimal complications [9,10].

Rodriguez et al. demonstrated good clinical results in a cohort of patients who underwent a novel fusion technique that uses CBT fixation in a previously instrumented pedicle with intraoperative O-arm guided navigation [11]. To the best of the authors' knowledge, no cases of robot-assisted CBT screw placement have been attempted in the dual trajectory fashion. Here we present a novel application of CBT fixation utilizing robotic guidance in a previously instrumented spine with a traditional PS.

## Case presentation

History

The patient is a 60-year-old woman with a history of prior left L3-L4 extreme lateral interbody fusion secondary to underlying spondylolisthesis and instability. Unilateral percutaneous PSs were placed on the left side at L3 to L5. Her symptoms resolved postoperatively.

The following year the patient returned to the clinic with progressive bilateral S1 radiculopathy and intolerable mechanical back pain. She completed six months of physical therapy in the past without relief. She denied any focal weakness, sensory changes, or bowel/bladder incontinence.

Physical examination

Motor examination demonstrated 5/5 strength globally. The patient experienced diminished sensation in the S1 dermatome. Otherwise, the sensation was intact to pinprick and light touch in all extremities. Great toe proprioception was intact bilaterally. Reflexes were 2+ throughout.

Neuroimaging

**Figure 1:**
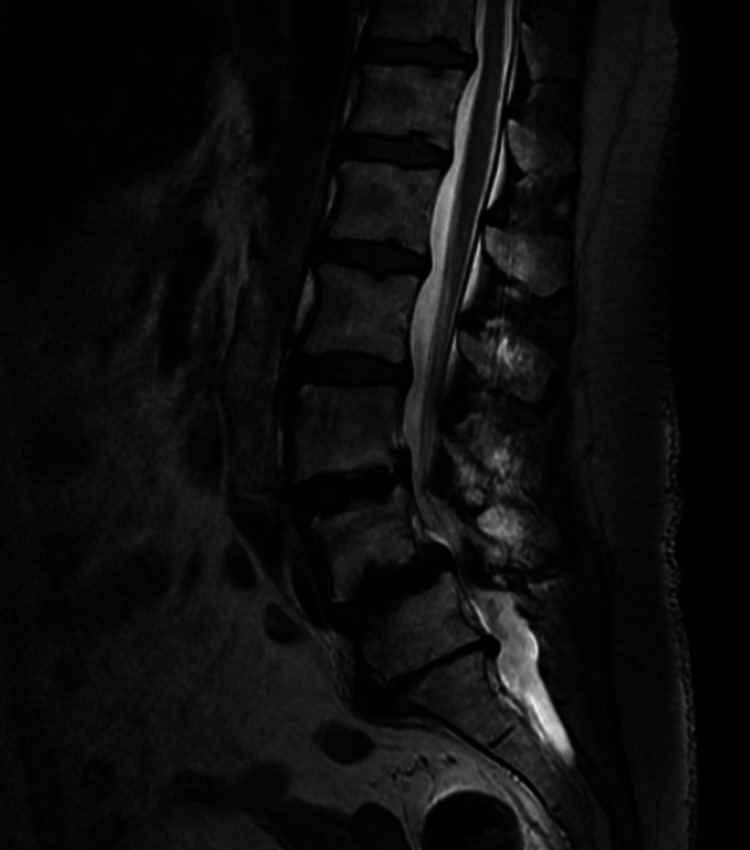
Preoperative lumbar MRI demonstrating L5-S1 disc bulge with disc collapse and modic changes at L5 and S1 loss of segmental lordosis secondary to ASD. MRI, magnetic resonance imaging; ASD, adjacent segment disease.

Lumbar magnetic resonance imaging (MRI) demonstrated L5-S1 disc bulge with disc collapse and modic changes at L5 and S1 with foraminal stenosis at L5-S1 and loss of segmental lordosis secondary to ASD (Figure 1). There were prior unilateral PS from L3-L5 on the left with lateral interbodies placed from L3-L4 and L4-L5. Imaging demonstrated bony fusion from L3-L5.

Operation

**Figure 2:**
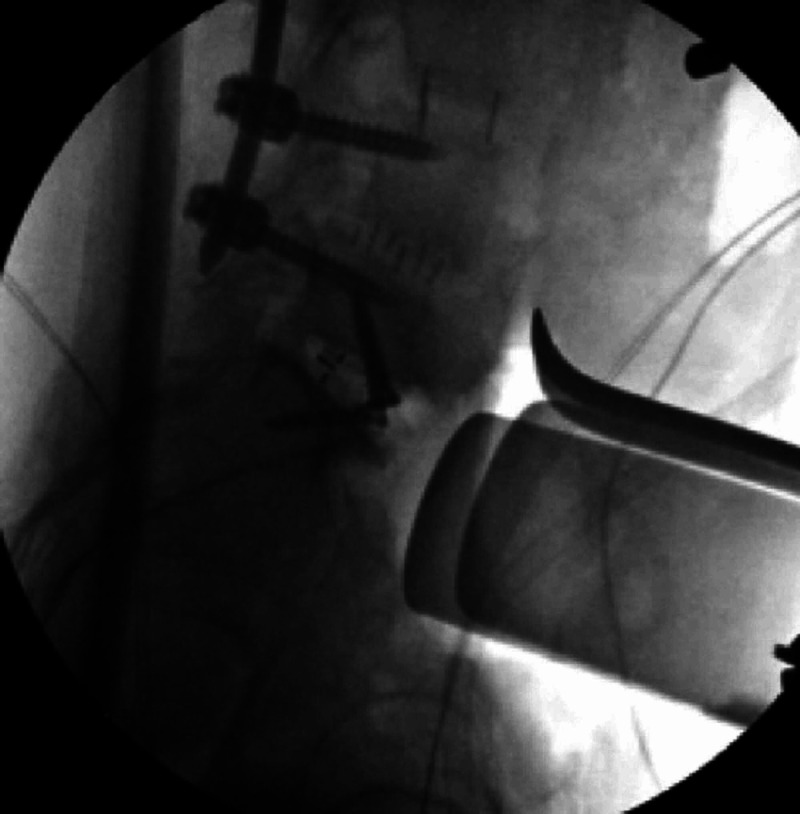
Intraoperative imaging following placement of the anterior interbody with the restoration of segmental lordosis and indirect foraminal decompression

**Figure 3:**
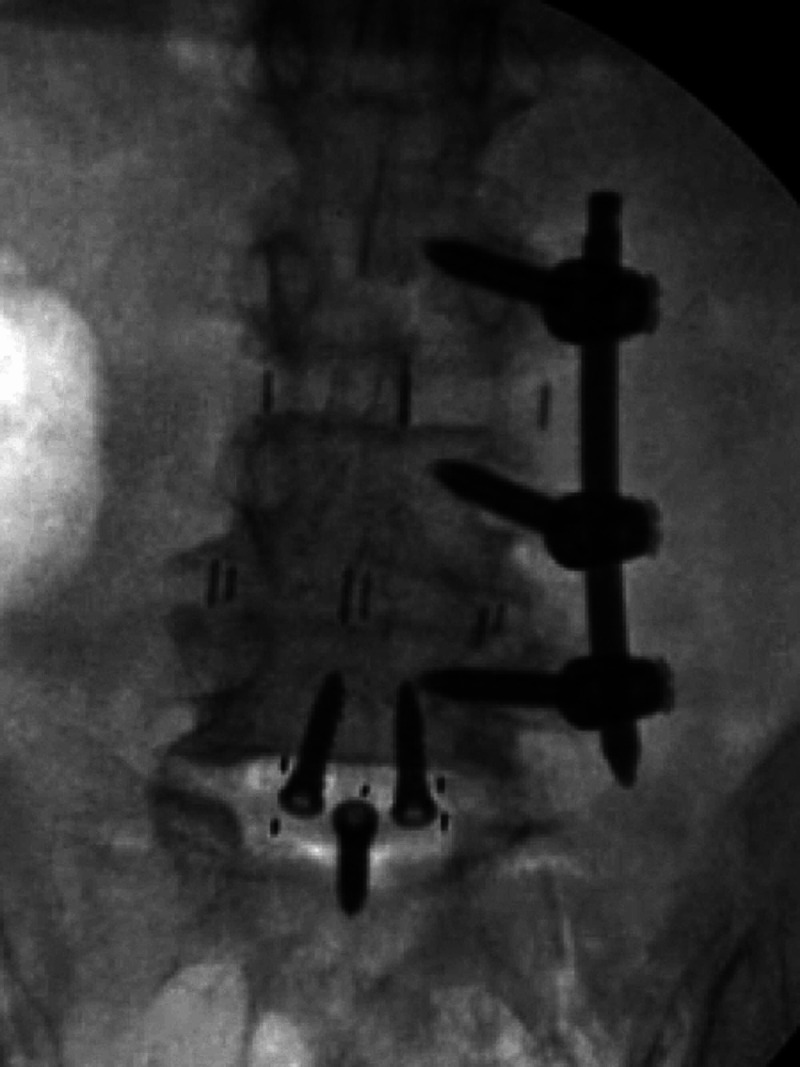
Anterior-posterior X-ray following placement of the L5-S1 anterior interbody demonstrating unilateral posterior instrumentation from L3-L5 on the left side

The patient underwent an L5-S1 anterior lumbar interbody fusion for indirect decompression and restoration of segmental lordosis (Figure 2, Figure 3). After the first stage was completed, she was turned prone for posterior percutaneous instrumentation. As there was no prior instrumentation on the right side, she underwent a percutaneous L4-S1 PS placement using the TP trajectory on the right side. Given the prior instrumentation at L3-L5 on the left side, the Mazor X Stealth Edition (Medtronic, Denver, CO, USA) software was used to plan CBT on the left L5 pedicle. A left S1 PS was then planned with the screw head aligning with the left L5 cortical bone screw.

**Figure 4:**
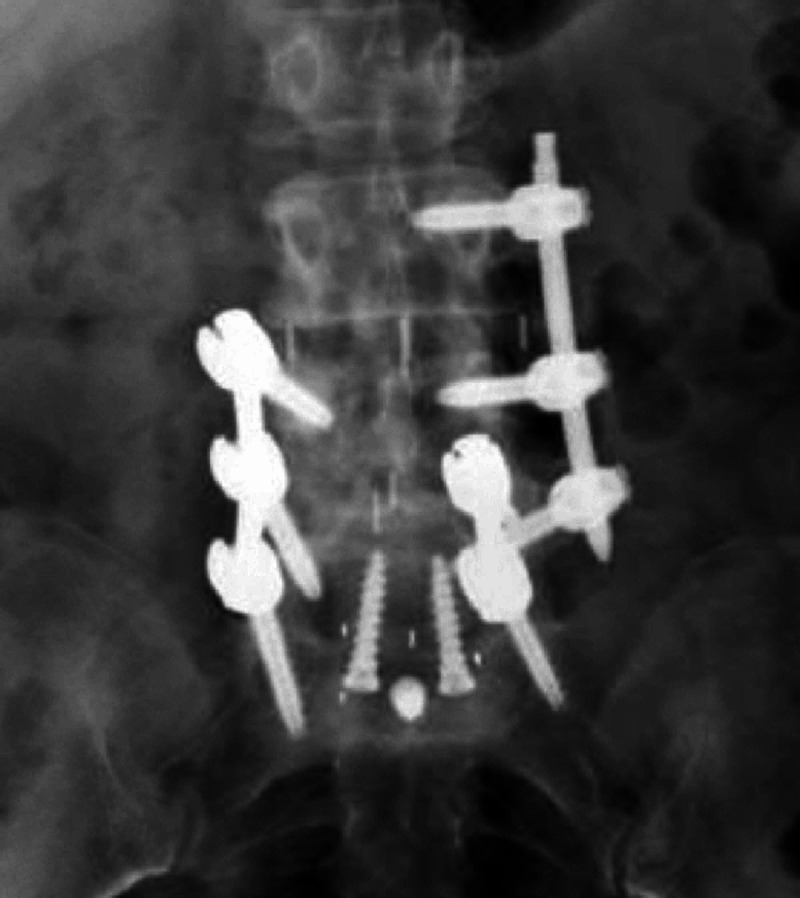
Postoperative anterior-posterior X-ray demonstrating right-sided traditional pedicle posterior instrumentation from L4-S1 on the right side. On the left side is an additional posterior construct at L5-S1 with a cortical bone trajectory screw at the same level as a traditional pedicle crew in the left L5 pedicle

**Figure 5:**
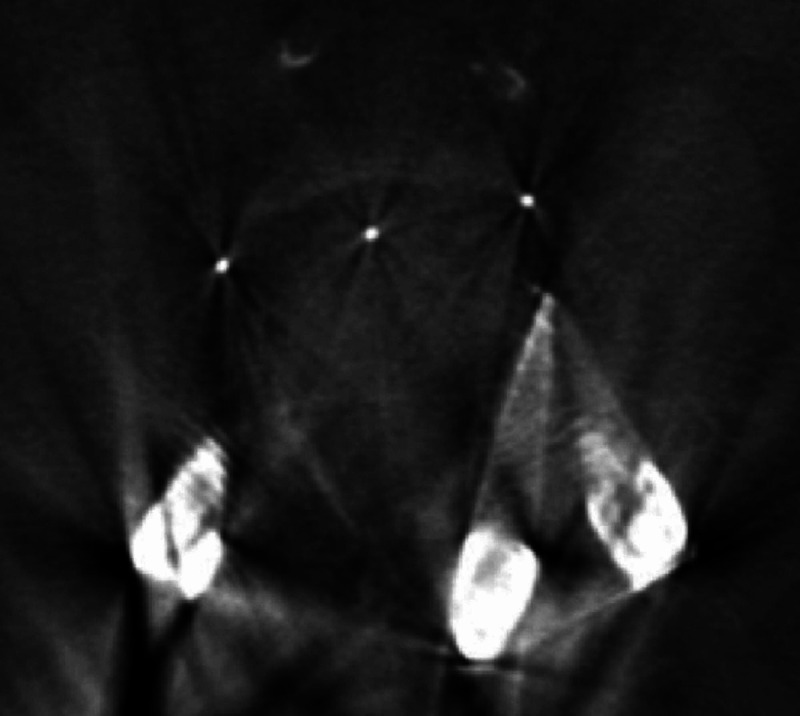
Postoperative axial computed tomography demonstrating the presence of a cortical bone trajectory screw in the same pedicle as a traditionally oriented screw

Percutaneous screws were placed bilaterally using the robotic platform without complications. Intraoperative fluoroscopic imaging and postoperative CT demonstrated accurate placement of PS (Figure 4, Figure 5).

Postoperative course

The patient had an uneventful three-day hospital stay, and her symptoms improved significantly. She was discharged home without physical therapy.

## Discussion

CBT screws are emerging as an alternative for instrumenting the lumbar spine. Khan et al. noted that when comparing CBT between robot technology and CT-navigation-guided cohorts, there were no significant differences in operative time, fluoroscopy time, and radiation dose. Another study found robot-assisted screw placement to be more accurate and safer compared with fluoroscopy-assisted placement for lumbar spinal CBT instrumentation [9].

Overall, the use of robotics in spine surgery has become more commonplace since it was approved by the US Food and Drug Administration in 2004 [12]. Robotic spine surgery can potentially enhance minimally invasive procedures by increasing accuracy and reducing radiation exposure compared to its standard counterpart procedure [13,14]. While current use is largely restricted to spinal fusion and instrumentation procedures, the feasibility of using robot technology to place a CBT screw at the same level as a traditional screw has not been well studied.

While there are various interventions to address ASD, surgical treatment to expose and remove previous instruments can result in significant postoperative pain, muscular fibrosis, and poor wound healing and infection. CBT fixation can mitigate some of these disadvantages and possibly obviate the need for hardware removal [3]. Prior studies have emphasized the importance of CT navigation for accurate CBT screw placement at levels where previous traditional PSs were already placed for symptomatic ASD [3].^ ^This technique is gaining popularity, as Chen et al. noted the minimal complications associated with this procedure [5].

The decision to utilize a robotic approach, in this case, was multifaceted: proven consistency, ease of operative planning, and rigidity of the Mazor system. Since Pechlivanis et al. published the first paper describing the robotic placement of percutaneous PSs, subsequent studies have demonstrated the high level of accuracy of spinal surgical robotics [15]. In one such study, O’Connor et al. report placing their first 90 PSs with 100% grade A accuracy on the Gertzbein-Robbins scale without any complications [14]. The Mazor software is able to segment each level of interest, allowing the operator to plan instrumentation along the optimal trajectory by viewing the screw placement in all three dimensions [14]. By doing so, Hyun et al. note the decrease in the tendency to violate the suprajacent facet with the robotic technique, reducing the likelihood of developing ASD [16]. Lieberman et al. illustrate the rigidity of the Mazor system, which attaches to the patient’s skeletal anatomy, providing an additional layer of security through a solid platform [17]. This allows the surgeon to register and reference images with a higher degree of accuracy compared to other robotic systems that do not have this anchoring capability.

Using the method described in this paper, a minimally invasive percutaneous approach was used to place posterior instrumentation following an anterior interbody. This technique obviated the need for a larger posterior incision, reducing the risk of infection, muscular dissection, and likely decreasing her hospital length of stay.

## Conclusions

This report is the first documented case of a robotically placed CBT screw placed in the same pedicle as a prior traditional PS for ASD. This method expands the surgical options for ASD to include percutaneous placement of PSs at the same level as previous instrumentation.
